# Reshaping daily life: An observational study on the impact of rheumatoid arthritis on occupational repertoire

**DOI:** 10.1177/03080226251404421

**Published:** 2025-12-27

**Authors:** Pedro H.T.Q. de Almeida, Tatiana Barcelos Pontes, Jane Davis, Lilian Dias Bernardo, Licia Maria Henrique da Mota

**Affiliations:** 1 University of Northern British Columbia, Prince George, BC, Canada; 2 Northern Center for Clinical Research, Prince George, BC, Canada; 3 University of Toronto, Toronto, ON, Canada; 4 Federal Institute of Rio de Janeiro, Rio de Janeiro, Brazil; 5 University of Brasilia, Brasília, Brazil

**Keywords:** Rheumatoid arthritis, occupational repertoire, participation, acs: activity card sort, occupational therapy

## Abstract

**Introduction:**

Rheumatoid arthritis (RA) restricts function and limits participation in everyday activities. While functional impairments are well-documented, RA’s impact on occupational repertoire, particularly in leisure activities, remains underexplored.

**Objective:**

To examine pre–post changes in occupational repertoire following RA onset and explore associations with functional status and disease activity.

**Methods:**

In this cross-sectional study, 32 adults with RA (28 women) from a tertiary rheumatology clinic completed the DAS-28, HAQ-DI, and Activity Card Sort – Brazil (ACS-BR) rating changes in their occupational repertoire before and after disease onset.

**Results:**

Post-onset participation fell in all ACS-BR domains (*p* < 0.01), most sharply in Instrumental (*r* = 0.73), High-Demand leisure (*r* = 0.75), and Social activities (*r* = 0.63), with an ICF mapping showing increased participation in health-management tasks (*p* < 0.01, *r* = 0.40). Functional disability, not disease activity, correlated with reduced Instrumental (ρ = −0.57) and Social (ρ = −0.39) participation.

**Conclusion:**

RA causes broad occupational disruptions, beyond self-care and productivity activities. Routine mapping of occupational repertoire can help occupational therapists detect early withdrawal from leisure and social roles, set client-centered goals, and monitor outcomes. Interventions that protect leisure and social participation, alongside self-management, may help sustain meaningful participation and quality of life.

## Introduction and Literature Review

Rheumatoid arthritis (RA) is a chronic, systemic, autoimmune condition characterized by persistent, bilateral, and symmetric joint inflammation and pain (Di Matteo et al., 2023). Although it can affect individuals of any age, incidence peaks during the fourth to fifth decades of life, with women affected two times more often than men. RA’s global prevalence is estimated at 0.25% to 1% of the general population, with higher rates reported among Indigenous and First Nations peoples (Granados et al., 2023). Despite significant advancements in pharmacological treatments, particularly with the advent of biologic therapies in the last decades, RA continues to impose a substantial burden on individuals, families, communities, and healthcare systems worldwide (Di Matteo et al., 2023; Gikaro et al., 2022).

Biomechanically, RA predominantly affects small, peripheral joints, particularly in the hands, resulting in restricted mobility, limited hand function, and dexterity (Di Matteo et al., 2023; Radu and Bungau, 2021). Such physical impairments translate into limitations across all areas of occupational performance, from activities of daily living (Iversen et al., 2017), work (Xavier et al., 2019), leisure (Strand et al., 2015; Veldhuijzen van Zanten et al., 2015), and social participation (Belau et al., 2024; Gikaro et al., 2022; Radu and Bungau, 2021).

The progressive course of RA impacts family dynamics (Zhao et al., 2018), employment status (Kirkeskov and Bray, 2023), and overall quality of life (Fairley et al., 2021; Radu and Bungau, 2021). These effects, in turn, diminish self-efficacy and social participation (Yan et al., 2024), and are often accompanied by the neglect of meaningful occupations observed among patients (Iversen et al., 2017). Ultimately, RA leads to the reshaping of individuals’ occupational repertoires, defined as the range and variety of activities and social roles which individuals do throughout their lives (Davis and Polatajko, 2010).

The onset of chronic conditions, such as RA, inevitably alters the occupational repertoire, prompting individuals to modify, reduce, or discontinue specific activities—often affecting both personal fulfillment and social connectedness. While research on the occupational participation of individuals with RA has expanded considerably in recent decades (Gavin et al., 2024; Sheerin et al., 2024), its focus remains concentrated on urban populations within high-income countries (Gavin et al., 2024). Consequently, there is a notable gap in the literature regarding changes in occupational repertoire among individuals with RA in non-Western settings.

As occupational repertoires are socially situated (Davis and Polatajko, 2010), sociocultural and economic contexts shape which activities are retained, modified, or discontinued through influences on activity choices, caregiving responsibilities, community involvement, and access to resources (Hammell, 2020). In Latin American countries, socioeconomic inequities may limit access not only to health care but also to protective factors for musculoskeletal health, including regular exercise, adequate nutrition, and social welfare supports. Coupled with cultural expectations and social norms, these inequities are compounded by the demands of double- or triple-shift work that combines paid employment with housework and caregiving, increasing the risk of occupational imbalance and disruption when chronic conditions arise (da Silva Pontes et al., 2024).

Recognizing these cultural distinctions is essential for developing client-centered, occupation-based interventions that address the unique factors shaping participation across different contexts.

## Objectives

As a primary objective, this study aimed to explore how RA alters the occupational repertoire of Brazilian adults, using a retrospective pre–post comparison of participation in daily activities before versus after disease onset.

Secondary objectives were (a) to identify which ACS-BR domains (e.g., Instrumental, Low and High-demand Leisure, Social) are most affected by RA; and (b) to evaluate associations among functional status, disease activity, and changes in occupational repertoire.

Our *a priori* exploratory hypotheses were as follows: (H1) Adults with RA report restricted occupational repertoire after disease onset, reflected by decreases in repertoire breadth (number of activities retained), and (H2) higher disease activity and worse functional status are associated with greater limitations in the occupational repertoire.

## Method

### Study design

Cross-sectional, observational study.

### Setting

The study was conducted between April 2019 and August 2019 at outpatient rheumatology clinics located at the University Hospital of Brasilia, a tertiary public teaching hospital in mid-western Brazil. Although the hospital is located in a major urban center, it serves a broad geographical area—including both urban and rural communities—in the mid-west, northeast, and northern regions of the country, providing specialized rheumatology care to a diverse patient population.

### Participants

A convenience sample of adults with RA was recruited from two rheumatoid arthritis clinics within the outpatient service. Adults aged 18 years and older were invited to participate if they met the following criteria: a formal diagnosis of RA according to American College of Rheumatology (Aletaha et al., 2010), treatment with disease-modifying antirheumatic drugs for at least six months, cognitive stability (able to provide informed consent, no history of significant cognitive impairment), and no history of neurological conditions affecting daily activities (e.g., stroke, traumatic brain injury, dementia, peripheral neuropathy) or acute musculoskeletal injuries (e.g., trauma, fractures, sprains) in the past year. Individuals with limited proficiency in written and spoken Portuguese, or those with temporary or permanent sensorimotor impairments (e.g., blindness, low vision, hearing loss, paresthesia), were excluded from the study. At the time of recruitment, all potential participants had been regularly followed by the clinic’s multiprofessional team for at least 1 year.

No a priori sample size calculation was performed; given the exploratory design, we opted to enroll all eligible participants during the study period.

Our study adhered to the Declaration of Helsinki and complied with all relevant national and institutional guidelines. Ethics approval was obtained from the Institutional Review Board of the Faculty of Health Sciences at the University of Brasília (protocol no. 845.114) prior to data collection. and all participants provided written informed consent before taking part in the study. Participation was strictly voluntary and took place outside of clinical appointments, and individuals were informed they could withdraw from the study at any time without consequences. No compensation for participation was offered, and all personal data were kept confidential and de-identified in the final dataset.

Participants were recruited through posters and flyers in waiting rooms and common areas throughout the hospital, as well as through direct referrals by clinic staff during regular appointments. Of 54 eligible individuals approached, 33 consented in participate; one participant did not complete the full assessment and was excluded from the study.

### Variables

#### Occupational repertoire

Changes in occupational repertoire were measured using the Activity Card Sort – Brazil (ACS-BR), a standardized tool with physical cards depicting 83 activities across four categories: instrumental activities, low-demand leisure activities, high-demand leisure activities, and social activities (Bernardo et al., 2020). In alignment with the original version, the ACS-BR considers instrumental activities as those related to community living and household maintenance (e.g., shopping, paying bills, attending medical appointments, using public transport); low-demand leisure encompasses recreational activities with minimal physical effort (e.g., reading, drawing/painting, cooking as a hobby), while high-demand leisure comprises more physically active pursuits (e.g., running, cycling, playing sports); last, social activities reflect interactional participation (e.g., spending time with friends & family, traveling, volunteering). The ACS-BR has shown excellent internal consistency (Cronbach’s α = 0.91), similar to the United States, the United Kingdom, Australia, Hong Kong, and Israeli versions of the instrument (Bernardo et al., 2021).

#### Functional status

To measure the current functional status of participants, we used the Health Assessment Questionnaire – Disability Index (HAQ-DI), a validated and widely used instrument (Fries et al., 1980) in its Brazilian Portuguese version (Ferraz et al., 1990). The HAQ-DI quantifies the difficulty experienced by individuals living with rheumatic conditions during the performance of actions and activities across 8 domains (dressing and grooming, arising, eating, walking, hygiene, reach, grip, and common daily activities), with scores ranging from 0 (*“no difficulty”*) to 3 points (*“unable to do”*). The Brazilian Portuguese version of the HAQ-DI has excellent reliability, with a test–retest correlation of 0.905 and interobserver correlation coefficient of 0.830 (Ferraz et al., 1990).

#### Disease activity

Disease activity was measured using the Disease Activity Score-28 (DAS-28) (Prevoo et al., 1995), which incorporates tender and swollen joint counts, patient global assessment, and inflammatory markers. DAS-28 scores range from 0 to 10, with higher values indicating increased disease activity. and participants’ disease activity was categorized as follows: Remission (<2.6), Low (2.6–3.2), Moderate (3.2–5.1), or High (>5.1) disease activity.

#### Data sources and measurement

Demographic data (age, sex, time since diagnosis) were collected from institutional records at the time of assessment.

To measure changes in the occupational repertoire, participants were asked to sort the ACS-BR cards into four categories, comparing their current occupational repertoire with the activities they performed before the onset of RA: (a) “*Activities that I used to do and still do*,” (b) “*Activities that I no longer do*,” (c) “*Activities that I started to do*,” and (d) “*Activities that I have never done and still don’t do*.” The ACS-BR was administered by three trained research assistants under the supervision of an experienced occupational therapist.

For each participant, three distinct scores were calculated for the ACS-BR and each of its four domains: (a) the number of activities currently performed, (b) the number of activities previously performed, and (c) the percentage of activities retained since the onset of RA.

Additionally, we mapped each activity from the ACS-BR to their corresponding categories within the Activities and Participation component of the International Classification of Functioning, Disability and Health – ICF (WHO, 2001). Two trained reviewers independently performed the mapping to ensure consistency, with a 97.8% agreement on the initial revision. A Chi-Square test confirmed the statistical significance of the agreement (χ^2^(1369, 83) = 2992.43, *p* < 0.001) with a subsequent Cohen’s Kappa suggesting high inter-reviewer reliability, with a score of 0.974. Disagreements were limited to two activities and were resolved through collaborative discussion. Following the mapping process, the activities from the ACS-BR were reclassified into 18 ICF categories.

The HAQ-DI was administered as a pen-and-paper questionnaire, completed by participants at the beginning of their assessment and scored by an occupational therapist with 8 years of experience in rheumatology. In accordance with HAQ-DI guidelines, participants were instructed to specifically consider their functional performance over the past week prior to the assessment. DAS-28 scores were determined by a rheumatologist on the same day as the assessment.

Given the potential selection and recall biases inherent in patient-reported outcomes, we mitigated these through standardized administration procedures and assessor training; further limitations and their implications are noted in the discussion section.

#### Statistical methods

Descriptive statistics (mean, standard deviation, range) were used to summarize demographic and clinical data. Given a non-normal distribution confirmed by the Shapiro–Wilk test, we applied non-parametric statistics to examine changes in occupational repertoire and its correlation with functional performance and disease activity.

ACS-BR scores were analyzed as raw totals, representing the absolute number of activities performed by each individual, except for the retained activity level score, which was calculated as the percentage of activities currently performed divided by those performed prior to the onset of RA (Bernardo et al., 2020). Participation in activities coded under ICF categories was expressed as a percentage, reflecting the proportion of activities performed within each category relative to the total number of activities listed. For example, a participation score of 50% in “Recreation and Leisure” activities indicates that participants engaged in 50% of the activities listed within that category at a specific point in time, rather than an absolute activity count.

A Wilcoxon Signed-Rank Test was used to compare participants’ occupational repertoire across ACS domains and ICF categories before and after RA onset. Due to the limited sample size, analyzing differences in individuals’ occupational repertoire across all four levels of disease activity was not feasible. To address this, individuals with moderate and high disease activity were combined into a single category, resulting in three groups. Differences in occupational repertoire among these three groups were assessed using a Kruskal–Wallis Test, with post hoc pairwise comparisons conducted, when applicable, to identify specific group differences. Lastly, associations among occupational repertoire, functional status, and disease activity were assessed by Spearman’s correlation coefficient.

Effect sizes were calculated using appropriate metrics for each statistical test, with Cohen’s *r* used for the Wilcoxon Signed-Rank Test. Spearman’s correlation coefficient (*rho*) and Cohen’s *r* were interpreted based on Cohen’s conventions with small (0.1–0.29), medium (0.3–0.49), and large (⩾0.5) effect sizes. An alpha (*p*) level of 0.05 was considered for statistical significance. All analyses were conducted in SPSS version 29.0 (IBM Corp, Armonk, NY, USA).

## Results

### Demographics

Thirty-two adults (28 females) with a mean age of 50.8 years (*SD*: 13.2) participated in this study. Individuals had a mean disease duration of 8 years (*SD*: 6.8). Most participants (*n* = 13, 40.6%) were in disease remission at the time of assessment. Key demographic and clinical characteristics are summarized in Table 1.

**Table 1.** Demographic and clinical characteristics.

Characteristic	*n* (%)	Mean (*SD*^c^; *range*)
Sex	32 (female: 28; male: 4)	–
Age (years)	–	50.88 (13.2; 25–87)
Disease duration (yrs)	–	8.03 (6.79; 1–37)
HAQ-DI^a^ (score)	–	0.74 (0.85; 0–2.75)
DAS-28^b^ (categories)		
Remission	13 (40.6%)	–
Low Activity	8 (25.0%)	–
Moderate Activity	8 (25.0%)	–
High Activity	3 (9.4%)	–

a HAQ-DI = Health Assessment Questionnaire – Disability Index.

b DAS-28 = Disease Activity Score-28.

c SD = Standard Deviation.

### Occupational Repertoire and RA

The onset of RA significantly altered participants’ occupational repertoire, with most individuals discontinuing activities they had regularly performed before the disease. On average, participants ceased participation in approximately 13% of activities they had been performing prior to the onset of RA. The largest decreases were observed in High-Demand Leisure (Z = −4.22, *p* < 0.01), followed by Instrumental (Z = −4.13, *p* < 0.01), Low-Demand Leisure (*Z* = −3.45, *p* < 0.01), and Social domains of the ACS-BR (*Z* = −3.55, *p* < 0.01; see Table 2).

**Table 2.** Changes in occupational repertoire after the onset of rheumatoid arthritis: Activity card sort – Brazil scores.

Categories	Pre-RA^a^ Score (*SD*^b^)	Current Score (*SD*^b^)	Retained **% (*SD*)**	*p*-Value*	Effect size (*r*)*
Total Score	48.34 (11.26)	41.66 (12.23)	86.76 (16.8)	<0.01	0.71
Instrumental Activities	15.81 (2.18)	13.06 (3.81)	82.19 (19.2)	<0.01	0.73
Low-Demand Activities	15.41 (5.43)	14.28 (5.26)	93.1 (10.5)	<0.01	0.61
High-Demand Activities	5.12 (3.12)	2.56 (2.11)	51.04 (32.9)	<0.01	0.75
Social Activities	12.00 (3.14)	10.25 (3.96)	79.9 (30.4)	<0.01	0.63

* Wilcoxon Signed-Rank Test.

a RA = Rheumatoid Arthritis.

b SD = Standard Deviation.

When comparing changes through the ICF categories, most individuals reported a significant reduction in participation in recreation and leisure activities (*Z* = −3.98, *p* < 0.01), followed by caring for household objects (*Z* = −3.5, *p* < 0.01), and performing household tasks (*Z* = −3.33, *p* < 0.01). Similarly, participation in both paid (*Z* = −3, *p* < 0.01) and voluntary work (*Z* = −2, *p* = 0.04) significantly decreased after the onset of RA.

Interestingly, a significant, but moderate increase in participation in activities related to looking after one’s health was observed, given the increasing number of participants reporting attending appointments with healthcare providers and managing medications (*Z* = −2.64, *p* < 0.01). In contrast, no significant differences were found in participation in activities related to mobility, religion and spirituality, and family and intimate relationships, among others (see Table 3).

**Table 3.** Changes in participation – ICF^a^ categories.

International Classification of Functioning, Disability and Health Category	Pre-RA Mean % (*SD*^b^)	Post-RA Mean % (*SD*^b^)	*p*-Value*	Effect size (*r*)*
Acquisition of goods and services	100.00 (0.00)	81.25 (35.36)	0.01	0.56
Applying knowledge	75.00 (43.99)	84.38 (36.89)	0.08	0.12
Caring for body parts	87.50 (33.60)	78.13 (42.00)	0.08	0.25
Caring for household objects	46.25 (21.81)	31.88 (19.58)	<0.01	0.6
Carrying out daily routine	90.63 (29.61)	75.00 (43.99)	0.09	0.29
Economic life	76.56 (40.13)	76.56 (40.13)	1	0
Education	40.63 (49.90)	40.63 (49.90)	1	0
Family relationships	56.25 (50.40)	46.88 (50.70)	0.08	0.18
Household tasks	93.13 (10.91)	73.75 (25.62)	<0.01	0.5
Intimate relationships	59.38 (49.90)	56.25 (50.40)	0.31	0.1
Looking after one’s health	70.31 (39.88)	92.19 (18.45)	<0.01	0.4
Moving around using transport	66.41 (23.43)	61.72 (25.39)	0.10	0.15
Nonremunerative employment	46.88 (50.70)	34.38 (48.26)	0.04	0.18
Purposeful sensory experiences	25.00 (43.99)	25.00 (43.99)	1	0
Recreation and leisure	49.56 (16.38)	41.31 (15.57)	<0.01	0.65
Religion and Spirituality	87.50 (25.40)	84.38 (29.61)	0.15	0.1
Remunerative employment	81.25 (39.66)	53.13 (50.70)	<0.01	0.35
Using communication devices	79.69 (24.95)	79.69 (24.95)	1	0

a ICF: International Classification of Functioning, Disability and Health.

b SD = Standard Deviation.

* Wilcoxon Signed-Rank Test.

### Disease activity and performance of daily activities

We observed no significant changes between participants with different stages of disease activity for the total ACS-BR scores (H (2) = 3.76, *p* = 0.152), as well as Instrumental Activities (H (2) = 4.97, *p* = 0.08), High-Demand Leisure (H (2) = 2.5, *p* = 0.285), Low-Demand Leisure (H (2) = 2.67, *p* = 0.263), or Social Activities (H (2) = 2.66, *p* = 0.265). Similarly, when analyzing participation using ICF categories, there were no significant differences between individuals in RA remission and those with different levels of disease activity.

### Occupational repertoire, functional status, and disease activity

We noticed a strong, negative association between the HAQ-DI and total ACS-BR scores (Spearman’s rho = −0.53, *p* = 0.002), as well as the HAD-DI and the ACS-BR scores for Instrumental (rho = −0.57, *p* < 0.001). A moderate, negative correlation was also observed for the scores of the HAQ-DI and the ACS-BR Social domain (rho = −0.39, *p* = 0.02). No significant correlations were observed among age, years since diagnosis, DAS-28, and the ACS-BR scores (see Table 4).

**Table 4.** Correlation among demographic, clinical, and occupational repertoire variables.

Variable	Age	Years since diagnosis	HAQ-DI^a^	DAS28^b^	ACS-BR^c^ Total Score	Instrumental Activities	Low-Demand Leisure Activities	High-Demand Leisure Activities	Social Activities
Age	.	0.54**	0.21	−0.03	−0.20	−0.13	−0.20	−0.03	−0.06
Years since diagnosis		.	0.22	0.06	0.03	−0.08	−0.03	0.24	0.09
HAQ-DI^a^			.	0.73**	−0.53**	−0.57**	−0.29	−0.34	−0.39*
DAS28^b^				.	−0.33	−0.33	−0.25	−0.25	−0.27
ACS-BR^c^ Total Score					.	0.83**	0.83**	0.60**	0.79**
Instrumental Activities						.	0.57**	0.41*	0.66**
Low-Demand Leisure Activities							.	0.44*	0.54**
High-Demand Leisure Activities								.	0.40*
Social Activities									.

Correlation coefficient represented by Spearman’s rho.

* *p* < 0.05. **p < 0.01.

a HAQ-DI = Health Assessment Questionnaire – Disability Index.

b DAS-28 = Disease Activity Score-28.

c ACS-BR: Activity Card Sort – Brazil

## Discussion

In this study, we examined how rheumatoid arthritis (RA) reshapes the occupational repertoire of Brazilian adults by comparing their participation in daily activities before and after disease onset. Our findings support our primary hypothesis (H1), showing a clear reduction in repertoire breadth—reflected in fewer retained activities and roles—across multiple domains. Consistent with our a priori hypothesis (H2), such changes were associated with decrease functional status, whereas associations with disease activity were less consistent, likely reflecting reduced statistical power after collapsing categories and the cross-sectional design.

The largest declines occurred in activities requiring higher-energy expenditure^1^—particularly those in the Instrumental and High-Demand Leisure categories of the ACS-BR, with many participants discontinuing their participation in sports, household maintenance, and work-related activities. These shifts were accompanied by notable reductions in social interactions and low-demand leisure activities, underscoring the broad and multifaceted impact of RA on daily life.

Pain, fatigue, and limited mobility are well-documented barriers to participation in physical activity and sports among individuals with RA (Veldhuijzen van Zanten et al., 2015). In Brazil and other Latin American countries, cultural attitudes and norms may further deprioritize sports and leisure activities—particularly among older adults (Larsen et al., 2015)—viewing them as nonessential. This view contrasts with prevailing perspectives in North America (Iversen et al., 2017) and Europe (Belau et al., 2024; Brodin et al., 2023), where participation in leisure and physical activity is more strongly encouraged, including for those with chronic health conditions. Such cultural attitudes may have intensified the decline in these activities among our participants, despite evidence of their physical and psychosocial benefits for individuals with musculoskeletal and rheumatic conditions. Beyond physically demanding pursuits, Low-Demand Leisure (e.g., reading, hobbies) and social participation also declined, suggesting that the need for energy conservation and symptom unpredictability may limit lower-effort, restorative occupations alongside higher-effort ones.

Nevertheless, despite such contextual differences, our findings are consistent with international literature documenting significant reductions in physically intense activities among adults with RA in both high-income and low- to middle-income countries (Brodin et al., 2023; Gwinnutt et al., 2023; Huang et al., 2024).

Due to the insidious nature of RA, often marked by a sudden onset between the fourth to fifth decades of life—a period when most adults are typically expected to maintain active roles as workers, caregivers, or community members—patients frequently face the challenge of prioritizing self-care, productivity, and caregiving responsibilities, such as caring for children or elderly parents, often at the expense of leisure and social activities (Ahlstrand et al., 2012; Yan et al., 2024). Moreover, cross-sectional studies utilizing nationwide data indicate that approximately 45% of individuals living with arthritis in the United States experience limitations in engaging in meaningful activities, with most restrictions concentrated in leisure and social activities (Gikaro et al., 2022). Although the same studies report that activities of daily living and work-related tasks were also affected, these domains appear to be less extensively impacted, suggesting a differential effect of RA across areas of participation.

In our study, approximately 35% of participants reported being unable to participate in work activities after the onset of arthritis. Although the observed work participation post RA was higher than in other studies with a similar population in Brazil (Gomes et al., 2018), our results reflect a global challenge: roughly one-third of individuals with RA leave the workforce within 2 to 3 years of diagnosis, and over half are no longer employed after 10 to 15 years (Kirkeskov and Bray, 2023). Sustained employment is known to benefit individuals with chronic musculoskeletal and rheumatic conditions by enhancing their mental health, financial stability, and treatment adherence (Gwinnutt et al., 2023). However, maintaining work remains a significant challenge, as most individuals with rheumatic conditions face substantial barriers due to the complex interplay of health-related limitations, workplace demands, and broader societal factors (Ahlstrand et al., 2012; Gomes et al., 2018; Hansen et al., 2017). These barriers can further exacerbate financial pressures, as RA imposes a considerable economic burden that may be even more pronounced in middle and low-income countries (Huang et al., 2024; Xavier et al., 2019). This point reinforces the need for systemic interventions ranging from individualized strategies, such as workplace accommodations, to broad societal-level initiatives that improve access to rehabilitation services and support systems for individuals with disabilities (Gwinnutt et al., 2023; Hansen et al., 2017; Xavier et al., 2019).

Beyond a significant reduction in participation across all activity domains, our findings suggest an important shift in the types of activities prioritized after the onset of RA; when analyzing changes in occupational repertoire through the ICF domains, many participants reported reduced participation in household maintenance (e.g., fixing objects, doing laundry), recreation, and work-related activities (both paid and unpaid). Simultaneously, participants reported a significant increase in health-management-related occupations, with activities such as medication management and attending medical or allied health appointments consistently reported by more than 90% of participants after the onset of RA.

Such a shift may reflect a necessary prioritization of activities related to disease management, often at the expense of leisure activities that contribute to personal fulfillment and broader social participation (Fairley et al., 2021; Strand et al., 2015). A similar trend was observed in a multinational survey involving 1958 women with RA from North America and Europe, which found that RA symptoms led to a loss of spontaneity in leisure activities and an inability to continue hobbies, underscoring the broader impact of the condition on performance and participation outside of essential daily tasks (Strand et al., 2015).

Although we observed an overall decrease in participation in social activities, our results did not indicate significant changes in participation in religious and spiritual activities, such as attending places of worship or engaging in religious ceremonies or personal practices like prayer and religious readings. Religious and spiritual practices play a crucial role in the lived experiences and coping strategies of individuals with chronic inflammatory conditions such as RA (Keefe et al., 2001). These practices are particularly important in Brazil and Latin America, where religious involvement is high among older women (Moreira-Almeida et al., 2010), a demography that comprised a significant portion of our sample. Continued participation in religious activities despite physical symptoms, such as pain, movement restrictions, and fatigue, suggests that these activities may be prioritized due to their perceived emotional and social significance (Rzeszutek et al., 2017).

Finally, participants in our study did not report significant changes in their intimate relationships after the onset of RA, which is somewhat surprising given that between 30% and 70% of people living with RA experience some degree of sexual dysfunction (Zhao et al., 2018). This discrepancy may be related to the nature of the questions presented by the ACS, both in its original and translated versions, where “*Being with Spouse or Partner*” is the closest item addressing intimate relationships. This item does not explicitly explore potential limitations in sexual or intimacy-related activities. Therefore, our findings should not be interpreted as an absence of sexual dysfunction among participants. Given the higher prevalence and that reduced social participation is associated with higher risk of sexual dysfunction (Tański et al., 2022), this null finding should be interpreted cautiously and argues for more specific measures in future work.

## Strengths and limitations

To our knowledge, this study is the first to investigate changes in the occupational repertoire of individuals with RA using the ACS, offering a valuable contribution by capturing occupation-specific data within the diverse sociocultural landscape of the Global South. By integrating functional and clinical assessments, our study provides a nuanced perspective on how these interactions may influence activity choices. However, several limitations must be considered when interpreting these findings. Due to its cross-sectional design, this study does not allow for causal inferences or longitudinal insights into the evolution of individuals’ occupational repertoires over time. Additionally, the use of a convenience sample and a moderate sample size, and the absence of socioeconomic and cultural data limit the generalizability of our results and constrain subgroup analyses. Last, an inherent limitation of the ACS and other self-reported measures is the potential for recall bias, particularly when participants were asked to retrospectively report pre-RA activities and routines, which may affect the accuracy of perceived changes in occupational performance and participation.

Despite these limitations, our findings provide critical insights into the occupational impact of RA, highlighting patterns of activity modification and potential areas for intervention. By addressing a gap in the literature and contextualizing occupational participation in an understudied population, this study lays the groundwork for future longitudinal research and targeted rehabilitation strategies aimed at optimizing activity participation and quality of life for individuals with RA.

## Conclusion

Our findings highlight the profound impact of RA on individuals’ occupational repertoires, suggesting a contraction of occupational repertoires after RA onset, with the largest declines in leisure and social activities and a relative shift toward health-related tasks. While cultural factors likely shape which occupations are relinquished or maintained, this pattern of reduced participation is consistent with the wider RA literature.

Addressing participation—not only impairment—is therefore essential. The association between functional status and occupational repertoire supports the use of repertoire changes as a meaningful metric for assessing RA treatment outcomes. Incorporating the ACS, or similar occupation-based tools, into routine practice allows occupational therapists to detect and address not only changes in the performance of basic activities typically assessed by functional evaluations, but also early participation withdrawal from leisure and social activities, facilitating the development of truly client-centered goals.

Early and sustained occupational therapy intervention is critical not only for preserving functional capacity but also for maintaining participation in meaningful roles. The expected increase in health-management activities after the onset of a chronic condition, such as RA, further reminds practitioners to help clients balance demanding healthcare regimens with meaningful goals, preventing medical routines from taking over leisure and social participation.

Future research—ideally longitudinal and multi-site—should track how occupational performance and repertoire evolve over time and test whether targeted interventions sustain or restore participation across diverse sociocultural contexts.

**Key findings**

- Rheumatoid arthritis onset reduced participation across a wide variety of activities and occupational roles.
- Participation in leisure activities, sports, household chores, and social functions fell markedly after initial symptoms.
- Functional status was strongly associated with reduced occupational repertoire, particularly for activities in the Instrumental and Social domains of the ACS-BR.

**What this study has added**

- Rheumatoid arthritis leads to measurable alterations in occupational repertoire, with marked declines in the Instrumental, High-Demand Leisure, and Social domains of the Activity Card Sort, suggesting that occupational disruption extends well beyond self-care and productivity roles.

## Notes

1. While High- and Low-Demand Leisure activities are ACS-BR domains, the term “*higher-energy*” is used here as a descriptive phrase that may cut across domains (e.g., heavy housework within the Instrumental domain, higher-demand sports activities such as swimming, running within activities in the High-Demand Leisure domain) and should not be interpreted as an ACS-BR or ICF category.
